# Mitochondrial quality control in health and cardiovascular diseases

**DOI:** 10.3389/fcell.2023.1290046

**Published:** 2023-11-06

**Authors:** Asli E. Atici, Timothy R. Crother, Magali Noval Rivas

**Affiliations:** ^1^ Department of Pediatrics, Division of Infectious Diseases and Immunology, Guerin Children’s at Cedars-Sinai Medical Center, Los Angeles, CA, United States; ^2^ Infectious and Immunologic Diseases Research Center (IIDRC), Department of Biomedical Sciences, Cedars-Sinai Medical Center, Los Angeles, CA, United States

**Keywords:** mitochondria, cardiovascular disease, mitochondrial dynamics, mitophagy, mitobiogenesis, ROS

## Abstract

Cardiovascular diseases (CVDs) are one of the primary causes of mortality worldwide. An optimal mitochondrial function is central to supplying tissues with high energy demand, such as the cardiovascular system. In addition to producing ATP as a power source, mitochondria are also heavily involved in adaptation to environmental stress and fine-tuning tissue functions. Mitochondrial quality control (MQC) through fission, fusion, mitophagy, and biogenesis ensures the clearance of dysfunctional mitochondria and preserves mitochondrial homeostasis in cardiovascular tissues. Furthermore, mitochondria generate reactive oxygen species (ROS), which trigger the production of pro-inflammatory cytokines and regulate cell survival. Mitochondrial dysfunction has been implicated in multiple CVDs, including ischemia-reperfusion (I/R), atherosclerosis, heart failure, cardiac hypertrophy, hypertension, diabetic and genetic cardiomyopathies, and Kawasaki Disease (KD). Thus, MQC is pivotal in promoting cardiovascular health. Here, we outline the mechanisms of MQC and discuss the current literature on mitochondrial adaptation in CVDs.

## 1 Introduction

**FIGURE 1:**
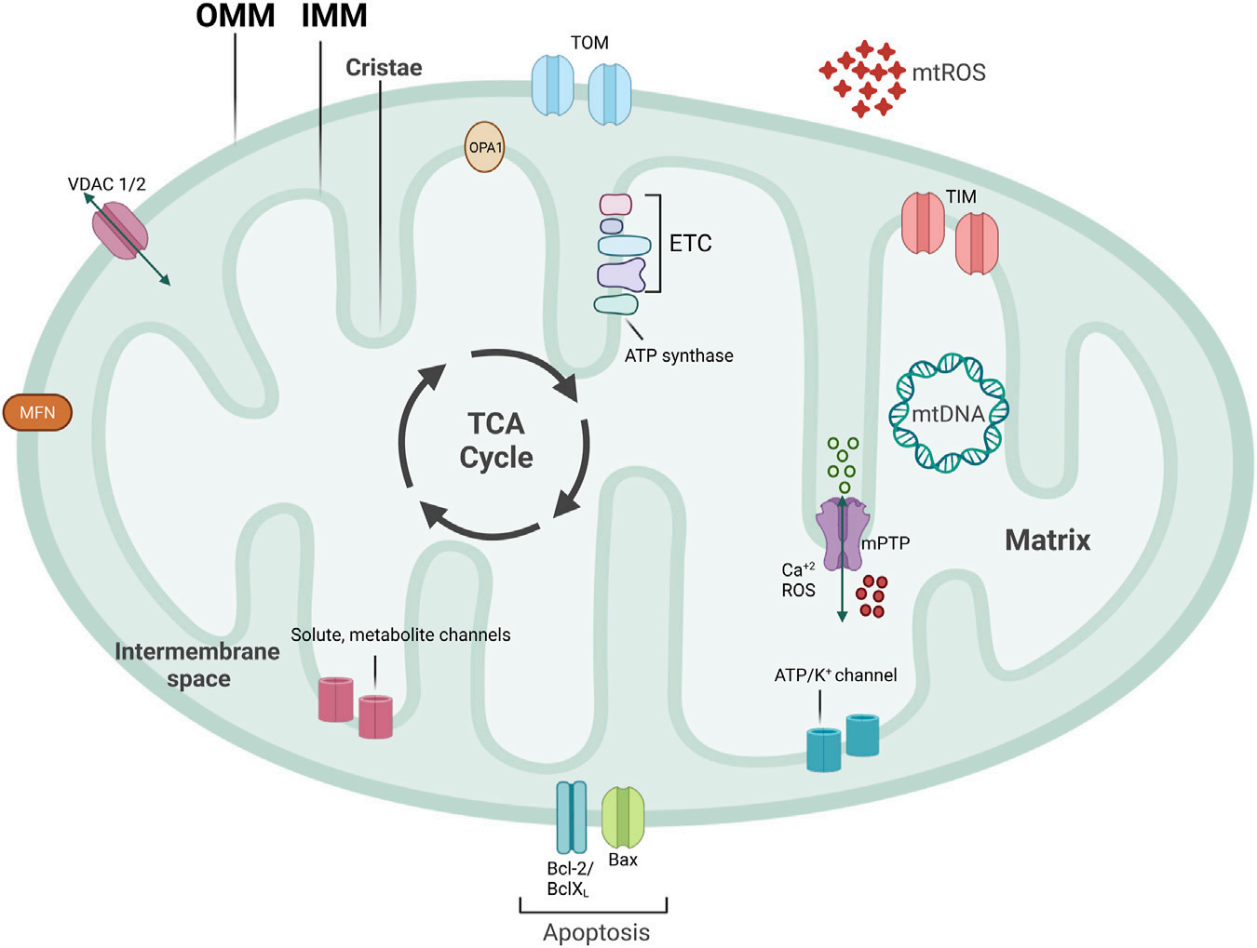
Mitochondrial structure. Mitochondria are double-membraned organelles with cristae formation. Mitochondria functioning is central for bioenergetic activities, producing ATP, an important source of ROS production. Mitochondria have several channels and pores to allow for metabolite and protein import, pivotal for inter-organelle communication. Part of ETC subunits are encoded in mitochondria’s circular DNA, named mtDNA. TCA cycle that produces important electron carriers for ETC, as well as ATP, and resides on the IMM. mtROS, mitochondrial reactive oxygen species; ETC, electron transport chain; mtDNA, mitochondrial DNA; IMM, inner mitochondrial membrane; OMM, outer mitochondrial membrane; mPTP, mitochondrial permeability transition pore; VDAC, voltage-dependent anion channel.

Mitochondria are central signaling hubs for eukaryotic cells involved in metabolism, inflammation, calcium regulation, and cell death (Figure 1) (Galluzzi et al., 2012; Lopez-Crisosto et al., 2017; Mehta et al., 2017; Galluzzi et al., 2018). Mitochondria are double membrane organelles consisting of an outer (OMM) and an inner mitochondrial membrane (IMM), both delimiting an intermembrane space (Figure 1). The IMM folds inwards, forming crests (cristae), which increase the surface area available for mitochondrial reactions (Protasoni and Zeviani, 2021) (Figure 1). Through the breakdown of carbohydrates and fatty acids and the production of adenosine triphosphate (ATP) via oxidative phosphorylation (OXPHOS), mitochondria are the primary source of energy for eukaryotic cells (Madeira, 2018). This process is mediated by products from the tricarboxylic acid cycle (TCA cycle) entering the mitochondrial electron transport chain (ETC), a group of proteins located in the IMM (Figure 1) (Martínez-Reyes and Chandel, 2020). Eleven ETC subunits are encoded in mitochondria’s circular DNA (mtDNA) and are crucial to correctly assembling these complexes (Vercellino and Sazanov, 2022). The remaining ETC subunits are encoded by the nuclear DNA and imported into the mitochondria (Yan et al., 2019) (Figure 1). Mitochondria are also involved in ROS production and communicate with other organelles, such as the endoplasmic reticulum (ER), via channels and pores, allowing metabolites and protein transport (Figure 1). Mitochondria are a highly dynamic network of organelles, strictly regulated by mitochondrial quality control mechanisms (MQC) (Ni et al., 2015). Due to cardiac tissues’ elevated energy requirement, maintenance of cardiovascular homeostasis is heavily dependent on mitochondrial quality. Indeed, cardiomyocytes utilize the ATP that mitochondria produce from carbohydrates and fatty acid-driven oxidative phosphorylation. Therefore, mitochondrial dysfunction affects cardiomyocytes’ function and contractility. Calcium levels are also central to cardiac activity. Calcium storage and regulation by mitochondria and the ER modulate cardiac function and impact electrical conduction (Lai and Qiu, 2020). Damaged or dysfunctional mitochondria are constantly eliminated via a mitochondria-specific degradation machinery, known as mitophagy (Sica et al., 2015; Harper et al., 2018). When mitophagy is dysregulated, damaged mitochondria accumulate inside cardiomyocytes, vascular smooth muscle cells (VSMCs), and endothelial cells, which can trigger inflammatory responses. Severe mitochondrial dysfunction and lack of clearance of damaged mitochondria can also initiate cell death and lead to tissue damage or loss (Hu et al., 2015; Amgalan et al., 2017; Gisterå and Hansson, 2017). Therefore, perturbations in mitochondrial quality promote the pathogenesis of CVDs, including ischemia/reperfusion, hypertension, atherosclerosis, heart failure, diabetic and genetic cardiomyopathies, and Kawasaki Disease (KD) (Bravo-San Pedro et al., 2017; Suomalainen and Battersby, 2018). Herein, we discuss several pathways related to mitochondrial metabolism and dynamics, how aged and dysfunctional mitochondria are eliminated, how new mitochondria are produced, and the importance of MQC to several CVDs.

## 2 Mitochondrial metabolism and function

### 2.1 Tricarboxylic acid (TCA) cycle

**FIGURE 2:**
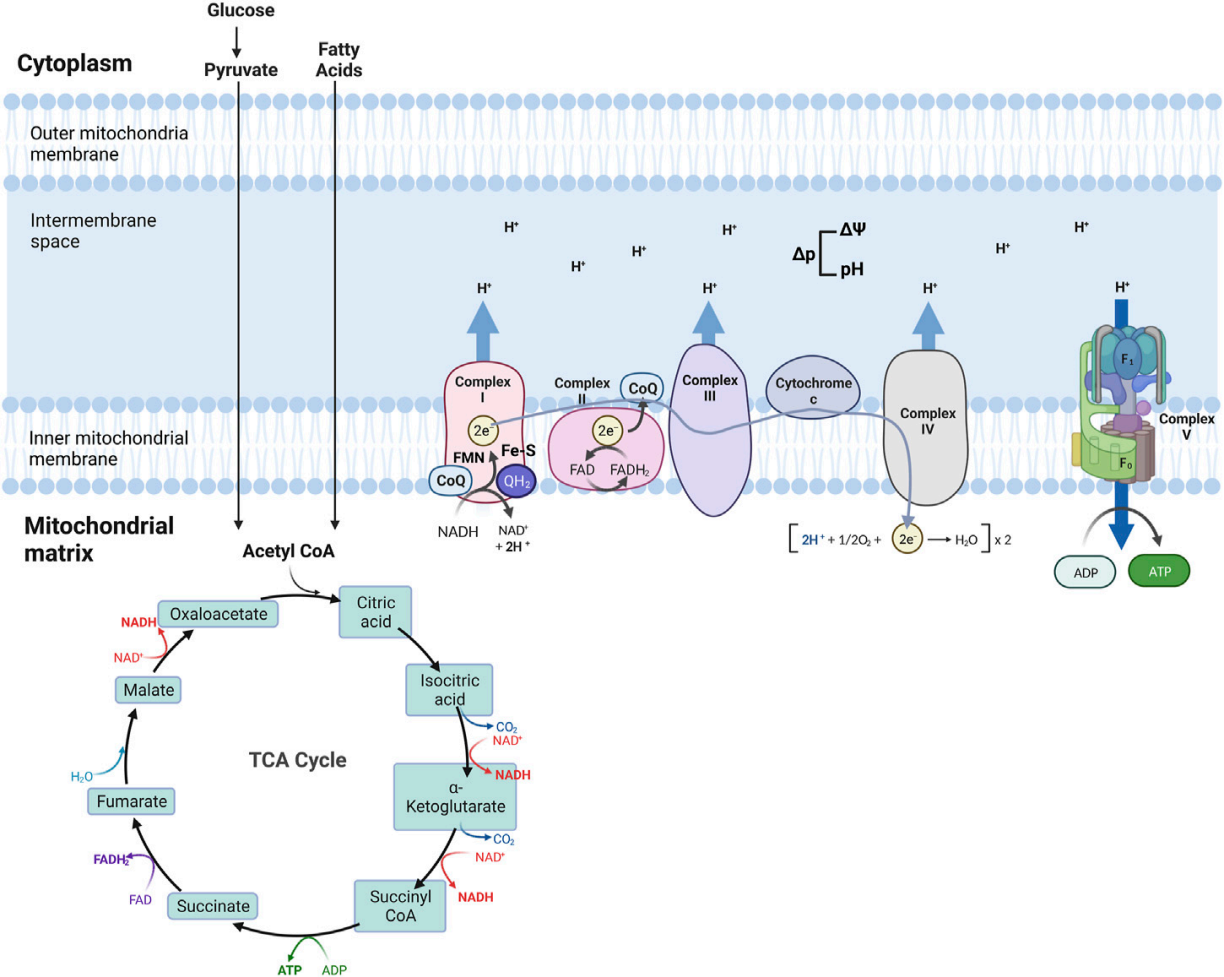
Schematic of mitochondria electron transport chain (ETC) and Oxidative phosphorylation (OXPHOS). ETC comprises four complexes that reside in the inner mitochondrial membrane that leads to the production of ATP. The TCA cycle is the source of NADH and FADH_2,_ which are required for electron transport between the complexes, finalized by the production of ATP at the last step.

The Tricarboxylic acid (TCA) cycle (citric acid cycle or Krebs cycle) comprises a series of reactions occurring in the mitochondrial matrix, involving numerous substrates crucial to cellular metabolism (Figure 1). Metabolites of the TCA cycle regulate cellular function. They are necessary for the biosynthesis of lipids, nucleotides, and proteins (McDonnell et al., 2016), as well as chromatin modifications, DNA methylation, and post-translational protein modifications (Wellen et al., 2009; Moussaieff et al., 2015). The TCA cycle is initiated by a reaction that produces six-carbon citrate from two-carbon acetyl-CoA. Acetyl-CoA is generated from the oxidation of amino acids, glucose, fatty acids, or pyruvate (Figure 2). Acetyl-CoA combines with a four-carbon oxaloacetate molecule to yield citrate, which initiates the TCA cycle (Figure 2). Then, the oxidative decarboxylation of citrate isomer, isocitrate, forms α-ketoglutarate, a five-carbon molecule. Conversion of α-ketoglutarate to succinyl-CoA, a four-carbon molecule, yields two carbon dioxide (CO_2_) and two nicotinamide adenine dinucleotide (NADH or NAD^+^) molecules. Succinyl-CoA is then converted into succinate-producing GTP, which can later be converted into ATP (Shi and Tu, 2015; Martínez-Reyes et al., 2016). Oxidation of succinate to four-carbon fumarate transfers two hydrogens to flavin adenine dinucleotide (FAD), forming FADH_2_ (hydroquinone form). Lastly, fumarate is converted to malate and oxaloacetate, forming acetyl-CoA to proceed with the cycle (Martínez-Reyes and Chandel, 2020) (Figure 2). As detailed in the next section, byproducts NADH and FADH_2_ from the TCA cycle are essential electron donors for mitochondrial OXPHOS (Figure 2).

### 2.2 Oxidative phosphorylation (OXPHOS)

Four transmembrane complexes form the ETC. These complexes use cytochrome c and ubiquinone to transfer electrons through the IMM, where the ETC is located (Figure 2). ETC complexes form hetero-, or homodimers called super-complexes to optimize their functioning (Iwata et al., 1998; Guo et al., 2017). Together with ATP synthase, these complexes form the OXPHOS system in the mitochondrial inner membrane (Figure 2).

The ETC is fueled by electrons supplied via by-products from the TCA cycle that are transferred to oxygen (O_2_), a process that also results in the formation of a proton gradient in the mitochondrial intermembrane space and the production of ATP (Martínez-Reyes et al., 2016). More specifically, byproducts of the TCA cycle, NADH and FADH_2,_ are electron sources for Complex I (NADH: ubiquinone oxidoreductase) or Complex II (succinate dehydrogenase), respectively (Figure 2). Transfer of electrons from NADH to Complex I is achieved by ubiquinone (CoQ) and requires co-factors flavin-mononucleotide (FMN) and iron-sulfur (FeS) clusters, where CoQ is reduced to ubiquinol (QH_2_). Transfer of electrons to Complex I promotes the pumping of four protons into the mitochondria intermembrane space by Complex I. Electrons from FADH_2_ are conveyed to CoQ, with the co-factor FeS, and ultimately transferred to Complex II. Electrons entering the ETC are transferred to Complex III (coenzyme Q: cytochrome c reductase) via the oxidation of QH_2_ and the activity of the 2Fe-2S cluster. Electrons are then transferred to Cytochrome c, which releases two more protons into the mitochondrial intermembrane space. Cytochrome c is a mobile electron carrier that cargoes electrons from Complex III to Complex IV (cytochrome c oxidase). Complex IV comprises three subunits: I, II, and III. Subunit II interacts with reduced cytochrome c, transferring electrons to subunit I. This transfer also causes O_2_ to bind to Complex IV, which is reduced to water. Complex IV pumps protons from the mitochondrial matrix; some of them go into the intermembrane space and contribute to the proton gradient, while the remaining protons are used to form water molecules. The use of O_2_ for this process is known as the mitochondrial respiration (Fernie et al., 2004; Guo et al., 2018; Zhao et al., 2019; Vercellino and Sazanov, 2022).

As a result of the electron transport process, ten protons are pumped from the mitochondrial matrix into the intermembrane space where a proton gradient is built, which is the basis for the mitochondrial membrane potential (ΔΨ) (Figure 2) (Zorova et al., 2018). As the last step of oxidative phosphorylation, ATP synthase uses the proton motive force (Δp), the combination of ΔΨ and proton concentration, to produce ATP. More specifically, Δp couples electron transport from the other complexes to the activity of ATP synthase. ATP synthase consists of an extra-membranous (F_1_) and a transmembrane (F_0_) domain, which functions with a rotational mechanism to produce ATP. At the last step of OXPHOS, protons pumped to the intermembrane space return to the mitochondrial matrix through F_0_, dispersing the proton gradient. This allows for the rotation of ATP synthase, which induces the addition of phosphate to ADP to form ATP (Figure 2) (Reid et al., 1966; Capaldi and Aggeler, 2002; Harrington et al., 2023).

### 2.3 Mitochondrial reactive oxygen species (ROS) production, detoxification, and mtDNA damage

The process of electron transfer between the ETC complexes could be more efficient. Protons may leak and migrate into the mitochondrial matrix, which results in incomplete coupling of O_2_ with no ATP production (Cheng et al., 2017). Electrons may also leak and exit the ETC before being converted into water from Complex I and III, resulting in reduced O_2_ and increased production of reactive oxygen species (ROS) (Cheng et al., 2017). Mitochondria are a significant source of ROS, which can damage mitochondria and result in cell death when produced at high concentrations or act as a signaling molecule when made at low concentrations (Murphy et al., 2016). ROS comprises superoxide anion (O_2_•^-^), hydroxyl radical (•OH), and hydrogen peroxide (H_2_O_2_). Reduction of O_2_ by one electron causes the formation of O_2_•^-^ and can be dismutated to H_2_O_2_ (Murphy, 2009; Cadenas, 2018). Complex II has also been shown to be involved in ROS production as it performs a reverse transfer of electrons from succinate to ubiquinone and back to Complex I, a ROS production site (Liu et al., 2002; Yankovskaya et al., 2003). Various other mitochondrial enzymes also contribute to mitochondrial ROS production. Oxidation of lipids by acyl-CoA dehydrogenase or glycerol-α-phosphate dehydrogenase yields mitochondrial ROS in the tissues (St-Pierre et al., 2002; Lambertucci et al., 2008). In addition, the TCA cycle enzymes pyruvate and α-ketoglutarate dehydrogenase also increase mitochondrial ROS production (Starkov et al., 2004).

Voltage-dependent anion channels (VDACs) are OMM proteins that form aqueous pores to exchange metabolites (Colombini et al., 1996). There are three VDAC isoforms (VDAC1, 2, and 3), which are very similar in sequence and are expressed in cardiac tissue cells but have diverse functions (Zinghirino et al., 2021). VDAC1 and 2 play a role in mitochondrial bioenergetics and mitochondrial apoptotic pathways (Ravi et al., 2021). Nuclear-encoded proteins are responsible for modulating the redox state, and VDACs can sense the imbalance in the redox state in the mitochondria (Storz et al., 2005). It has been suggested that VDAC3 is a sensor of mtROS and VDAC1 is involved in ROS-induced apoptotic pathways (Brahimi-Horn et al., 2015; Reina et al., 2016). VDAC1 also partakes in the translocation of O_2_•^-^ to the cytosol from the mitochondria (Han et al., 2003). Furthermore, VDAC3 has also been shown as a target of ROS produced by Complex III of the ETC in mitochondria isolated from rat hearts (Bleier et al., 2015). In support of this, mice lacking VDAC3 have elevated mtROS production after being fed a high-salt diet (Zou et al., 2018). Similarly, a recent study showed that the absence of VDAC3 in human cells results in elevated ROS production and an increase in the ROS scavenging system to cope with oxidative stress, hinting at the protective effects of VDAC3 during oxidative damage. Additionally, the same study showed that VDAC3 deletion suppresses mitochondrial biogenesis (Reina et al., 2022).

O_2_•^-^ can react with nitric oxide (NO) that produces reactive nitrogen species (RNS). NO is critical during inflammation and acts as a pro-inflammatory mediator by promoting neutrophil accumulation, downregulation of adhesion molecules, and upregulation of apoptosis at high concentrations (Albina et al., 1991; Lu et al., 1996; Shin et al., 1996). NO also boosts vasodilatation in the cardiovascular system and activates macrophages (Coleman, 2001).

Numerous enzymes manage ROS detoxification and convert these byproducts into less reactive forms (Dubois-Deruy et al., 2020). O_2_•^-^ is very reactive and exhibits a short half-life, as it is constantly converted to H_2_O_2_ by mitochondrial manganese superoxide dismutase (MnSOD). H_2_O_2_ is less reactive and can diffuse out of the mitochondria as a cytosolic messenger (Giorgio et al., 2007; Candas and Li, 2014). Catalases detoxify H_2_O_2_ (Sepasi Tehrani and Moosavi-Movahedi, 2018). These Fe-heme-containing enzymes catalyze hydrogen peroxide into water and oxygen, mainly in the cytoplasmic peroxisomes (Kirkman and Gaetani, 2007). Glutathione peroxidases (GPx) and peroxiredoxins (Prx) are also involved in the breakdown of hydrogen peroxide. Glutathione is a co-factor and an electron donor for GPx. Prx is mainly located in the mitochondria, and like GPx, it converts H_2_O_2_ to water (Forman et al., 2009; Murphy, 2009; Forkink et al., 2010; Hossain et al., 2015).

ROS production can affect mtDNA integrity and lead to mtDNA damage. ROS production site is close to mtDNA in the IMM (Sampath, 2014). Importantly, mtDNA replication occurs in an asymmetric route, where heavy strands are left single-stranded, causing spontaneous deamination on the exposed nucleotides (Tanaka and Ozawa, 1994; Reyes et al., 1998). ROS-induced mtDNA damage causes missense mutations, deletions, or base substitutions, jeopardizing mtDNA integrity and mitochondrial functioning (Richter et al., 1988; Yakes and Van Houten, 1997). Numerous mtDNA repair pathways are used by the mitochondria, which take advantage of genomic DNA repair mechanisms (Kazak et al., 2012; Stein and Sia, 2017). The base excision repair (BER) pathway is the primary mechanism for mtDNA repair. Overall, BER eliminates oxidized, deaminated, or methylated bases, single-strand breaks, and alkylation damage (Sykora et al., 2012; Krokan and Bjørås, 2013). BER is a multi-step process that involves 1) recognition and removal of the target bases, 2) removal of abasic site, 3) end processing/gap filling, and finally, 4) DNA ligation (Jacobs and Schär, 2012). DNA glycosylases recognize and remove damaged DNA, and mitochondrial glycosylases include Uracil DNA glycosylase (UNG), 8-Oxoguanine DNA glycosylase (OGG1), and Nei-like DNA glycosylase 1 (NEIL1) (Hu et al., 2005). When damaged mtDNA accumulates, this interferes with the OXPHOS functioning (Ma et al., 2020). Consequently, mitophagy pathways are activated to clear defective mitochondria.

### 2.4 mtDNA mutations

Mutations of mtDNA itself or nuclear genes that encode for mitochondrial proteins can lead to perturbations in mitochondrial functioning and cause mitochondrial diseases (Russell et al., 2020). These mutations can include point mutations and rearrangements in mtDNA. Point mutations can alter genes that encode proteins, transfer RNA (tRNA) or ribosomal RNA (rRNA) and are inherited maternally (Pereira et al., 2021). Rearrangements of mtDNA are usually deletions or duplications and can either be inherited maternally or occur *de novo* (Chinnery and Hudson, 2013). Mutations that arise on nuclear genes that encode mitochondrial proteins can be inherited as X-linked, autosomal dominant, or autosomal recessive. These mutations usually affect ETC-related nuclear genes, mitochondrial import machinery, mitochondrial translational factors, or CoQ_10_ biosynthesis (Coenen et al., 2004; MacKenzie and Payne, 2007; Potgieter et al., 2013). Various nuclear-encoded proteins are responsible for maintaining mtDNA, which controls the synthesis of mitochondria deoxyribonucleoside triphosphates (dNTPs) and mtDNA replication. Any mutations in these genes may lead to the depletion of dNTPs or deficiency in mtDNA replication, inducing a decrease in the overall levels of mtDNA (El-Hattab and Scaglia, 2013). These mutations result in disturbances in ETC assembly and functioning, ultimately leading to a deficiency in organ ATP production, especially in high-energy-demanding cardiac tissues (Chinnery, 1993). Furthermore, as the OXPHOS system is defective due to mutations of mtDNA or nuclear DNA, NADH accumulates, blocking the TCA cycle and favoring the production of lactate from pyruvate, resulting in elevated levels of lactate that interferes with proper cardiac tissue functioning (El-Hattab and Scaglia, 2013). Cells can have identical mtDNA (homoplasmy), as well as a combination of different types (heteroplasmy) (Pereira et al., 2021). Mutations that alter all copies of mtDNA are homoplasmic, whereas mutations that affect certain copies of mtDNA and cause co-existence of normal and mutant mtDNA are called heteroplasmic mutations (Ballana et al., 2008; Stefano et al., 2017). Mitochondria are randomly distributed to the daughter cells during cell division, causing heteroplasmic mtDNA mutations to be inherited by chance. Therefore, mutant mtDNAs can accumulate at different rates in different tissues. Hence, as the mutated mtDNA content increases, energy production decreases, enabling CVDs susceptibility (Saneto and Sedensky, 2013; Gonçalves, 2019).

## 3 Mitochondrial quality control (MQC)

**FIGURE 3:**
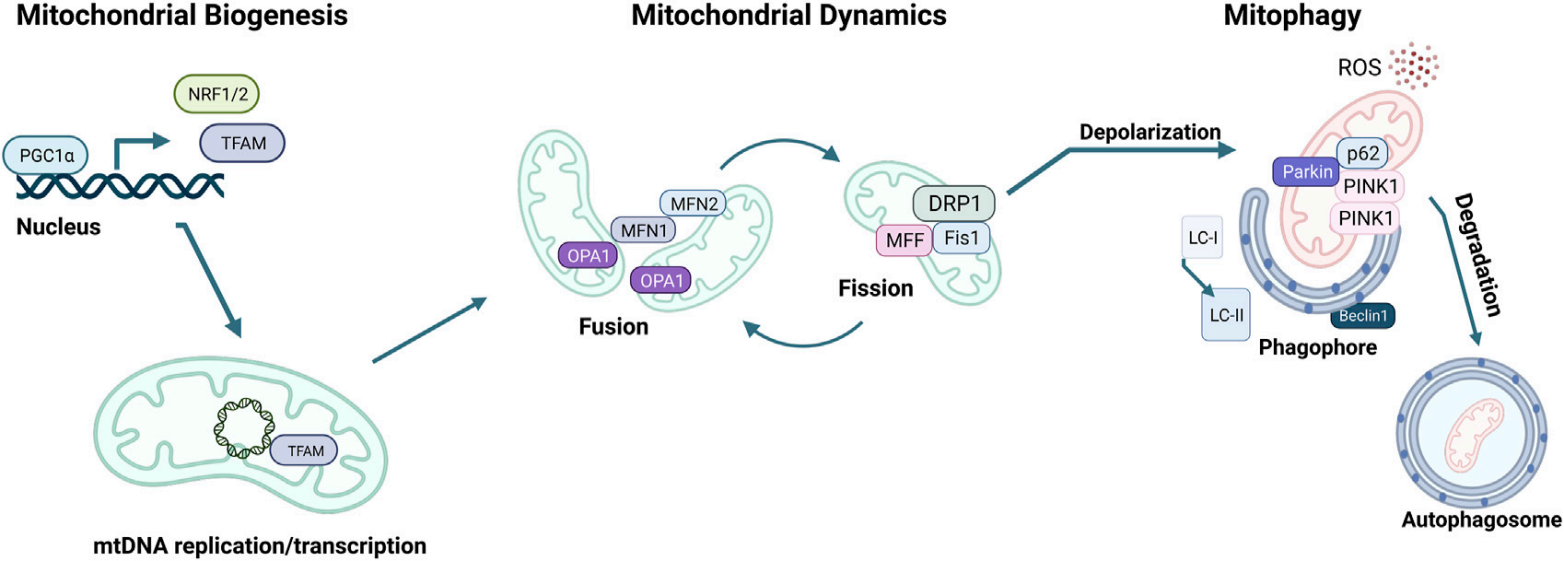
Mitochondrial quality control (MQC) mechanisms. The master regulator of mitochondrial biogenesis is PGC-1α, which induces NRF1, NRF2, and TFAM expression in the nucleus. Mitochondria life cycle includes fusion, which forms elongated mitochondrial networks, and fission, which creates fragmented mitochondria. MFN1, MFN2, and OPA1 are responsible for the membrane fusion of mitochondria, whereas DRP1, FIS1, and MFF mediate fission. Mitochondria accumulate oxidative damage during their expected lifespan or in the case of CVDs. Fission enables the fragmentation of damaged mitochondria to be separated and degraded. The elimination of damaged mitochondria is achieved via mitophagy, initiated by the accumulation of PINK1 kinase in the mitochondrial membrane, followed by the recruitment of Parkin, which targets the mitochondria to the autophagosome. p62 targets the mitochondria to the autophagosome and is eliminated during active autophagy. Assembly of the autophagosome requires Beclin1 and attachment of LC3-I onto phosphatidylethanolamine to form LC3-II.

MQC mechanisms are essential to maintain cellular mitochondrial homeostasis. MQC consists of a spectrum of various coordinated pathways. The primary function of MQC mechanisms is to ensure mitochondrial quality and maintain mitochondrial functions by limiting oxidative damage to mitochondria (Picca et al., 2018). ROS scavenging is a crucial first response to oxidative damage, and under physiological conditions, ROS production and detoxification are efficiently maintained (Lennicke and Cochemé, 2021). However, organelle damage can occur if ROS scavenging is insufficient to prevent a proper defense (Richter et al., 1995). ROS generation can also damage mtDNA due to the mitochondria’s inadequate DNA repair system (Druzhyna et al., 2008). At this point, a secondary line of defense is necessary for the mitochondria to continue normal functioning. These MQC mechanisms can be divided into two categories: molecular or organelle-level quality control mechanisms (Ng et al., 2021). Molecular level quality control includes the activation of mitochondrial chaperones, precisely heat shock protein 60 (HSP60), HSP70, and HSP90 (Bahr et al., 2022). These chaperones ensure proper folding of newly synthesized proteins and refolding of damaged ones before they are imported into the mitochondria (Becker and Craig, 1994). When misfolded or damaged proteins accumulate, they must be degraded via the ubiquitin-proteasome system (UPS). However, if the mitochondrial damage cannot be repaired via chaperones or UPS, organelle-level quality control mechanisms come into play, as summarized below (Kwon and Ciechanover, 2017; Jin et al., 2018) (Figure 3).

### 3.1 Mitochondrial biogenesis (mitobiogenesis)

The transcription and replication of the nuclear and mitochondrial genome are essential to mitochondrial biogenesis (mitobiogenesis), the process of self-replication of mitochondria. mtDNA encodes for 11 polypeptides of the ETC, as well as 22 tRNA and 2 rRNA (Fernández-Silva et al., 2003). Nonetheless, most of the mitochondrial proteins are encoded in the nuclear genome and transported into the mitochondria. For this reason, replication of mitochondria relies on mtDNA and nuclear DNA transcription to promote protein and lipid synthesis. Lipids, such as phosphatidylethanolamine (PE) or cardiolipin, are necessary for mitochondrial functioning and are made in the mitochondria but require ER-derived lipids (Tatsuta et al., 2014). Therefore, importing essential proteins and lipids from the nucleus or ER is critical for mitobiogenesis.

The master regulator of mitobiogenesis is the transcriptional coregulator peroxisome proliferator-activated receptor γ (PPARγ) coactivator 1α (PGC1α), which was first identified in brown adipose tissue as a coactivator of PPARγ (Puigserver et al., 1998). Numerous transcription factors are induced by PGC1α during external stimuli, such as exercise, cold, or fasting (Wu et al., 1999; Zong et al., 2002; Nisoli et al., 2005). Nuclear respiratory factors 1 and 2 (NRF1, 2) are two of such transcription factors, increasing the expression of mitochondrial transcription factor A (TFAM), which in turn translocates to mitochondria and induces mtDNA transcription (Virbasius and Scarpulla, 1994). Mitochondria resident micro-RNAs (miRs) also fine-tune mitobiogenesis by targeting TFAM or other mitobiogenesis factors, as well as relaying signals from the nucleus to the mitochondria to replenish the mitochondrial pool when necessary (Das et al., 2012; Das et al., 2014; Roman et al., 2020; Dogan et al., 2022). During mitobiogenesis, nuclear-encoded genes necessary for mitochondrial function are transcribed in the nucleus and translated into the cytosol with a mitochondrial localization signal. These proteins are transported to the mitochondria and are folded by mitochondrial chaperones (Wiedemann et al., 2004). Similarly, lipids are synthesized in the ER and transferred to the mitochondria during mitobiogenesis. This process involves the mitochondria-associated membranes (MAMs), which are mitochondria-ER contact sites (Gaigg et al., 1995).

Numerous external stimuli regulate PGC1α activity. Cold exposure and exercise induce PGC1α expression through β-adrenergic stimulation and cAMP response element-binding protein (cAMP/CREB) signaling (Wu et al., 1999; Baar et al., 2002). Moreover, AMPK activation in response to exercise also boosts PGC1α expression (Little et al., 2010). Posttranslational modifications are essential for the PGC1α activity (Miller et al., 2019). It has been shown that AMPK phosphorylates PGC1α on T177 and S538 in skeletal muscle (Jäger et al., 2007). Another crucial posttranslational modification on PGC1α is done by the NAD^+^-dependent deacetylase Sirtuin 1 (SIRT1). Deacetylation and activation of PGC1α by SIRT1 are essential, as NAD^+^ levels are highly dependent on AMPK activity. Therefore, in addition to its role in mitobiogenesis, PGC1α conjugates cellular energy metabolism, redox state, and mitochondrial activity (Gerhart-Hines et al., 2007; Coste et al., 2008; Cantó et al., 2009).

### 3.2 Mitochondrial fission-fusion

The mitochondrial network is highly dynamic, constantly remodeling through fission and fusion to decrease stress and replace damaged components (Parra et al., 2011; Chan, 2020). Fusion of two mitochondria is driven by dynamin-related GTPases mitofusin1 and 2 (MFN1, MFN2) on the OMM surface and optic atrophy protein 1 (OPA1) and cardiolipin on the IMM (Chen et al., 2003; Olichon et al., 2007; Paradies et al., 2019) (Figure 3). MFN1 also participates in ER-associated degradation (ERAD) through the degradation of damaged mitochondria via polyubiquitination by the E3 ubiquitin ligase Parkin (Picca et al., 2018). Mitochondrial fusion allows for the fusion of two mitochondria that are in close proximity to share mtDNA, metabolites, and enzymes. Fusion of the OMM requires homo- and heterodimerization of MFN1 and MFN2 (Figure 3) (Ishihara et al., 2004; Chen and Chan, 2009; Hall et al., 2014). OPA1 plays a role in the fusion of the IMM while preserving the cristae structure (Malka et al., 2005; Song et al., 2007). This fusion process sustains the mitochondrial membrane and its permeability to enhance protection from oxidative damage.

Damaged mitochondria can also undergo fission, where one mitochondrion divides into two mitochondria (Chen and Chan, 2009; Ong and Hausenloy, 2010; Elgass et al., 2013). Fission is orchestrated by GTPase dynamin-related protein 1 (DRP1), mitochondrial fission one protein (FIS1), and mitochondrial fission factor (MFF). FIS1 inhibits mitochondrial fusion and acts as a receptor for the binding of DRP1 to the OMM. Once recruited, DRP1 triggers cleavage of mitochondria via interacting with FIS1 and MFF (Palmer et al., 2011; Onoue et al., 2013; Otera et al., 2013; Atkins et al., 2016). Splitting off damaged mitochondria through fission preserves the healthy pool of mitochondria, optimal OXPHOS functioning, and efficient allocation of mitochondrial materials (Quiles and Gustafsson Å, 2022).

A balance between mitochondrial fission and fusion is critical for maintaining optimal mitochondria functioning at homeostasis or during pathological conditions, especially in CVDs. Clearance of damaged mitochondria is central to the control of mitochondrial quality. It is achieved through mitophagy, a process linked to fission since fragmented mitochondria are degraded via the autophagolysosomes (Zaffagnini and Martens, 2016). Mitochondrial fission occurs on the MAMs, where DRP1 also localizes and promotes nucleation of mitochondrial fission. ER tubules can also wrap around the mitochondria and cause constriction of mitochondrial membranes during fission (Friedman et al., 2011; Yang and Yang, 2013).

### 3.3 Mitochondrial clearance via autophagy/mitophagy

**FIGURE 4:**
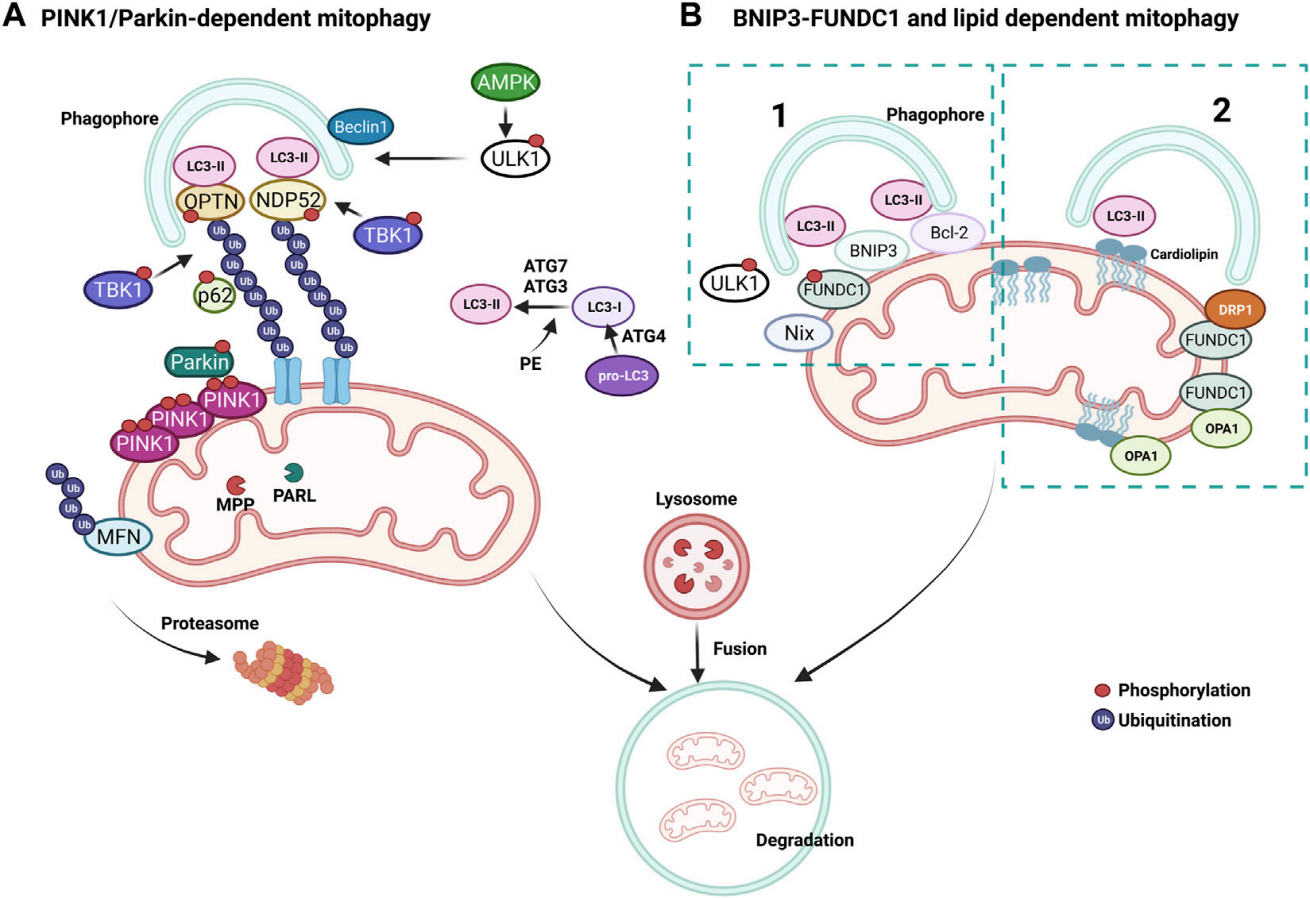
Mechanisms of mitophagy. **(A)** PINK1/Parkin-dependent mitophagy. PINK1 accumulates on damaged mitochondria and recruits Parkin to promote the ubiquitination of OMM proteins. Poly-ubiquitin chains provide an eat me signal to initiate autophagy. TBK1 phosphorylates OPTN and NDP52 to induce clearance of damaged mitochondria. **(B)** Parkin-independent mitophagy pathways, (1) BNIP3/FUNDC1 mediated and (2) lipid-mediated mitophagy. Mitophagy receptors BNIP3 and FUNDC1 favor mitochondrial fission via DRP1 interaction and OPA1 release.

A pivotal mechanism of MQC is a mitochondria-specific autophagy process named mitophagy (Figure 4). Clearance of mitochondria by mitophagy is indispensable during cellular differentiation, as well as for tissues that require high energy, such as the cardiovascular system (McWilliams et al., 2018). Mitophagy is needed to overcome stress in pathological conditions, and evidence from multiple studies suggests that a deficit in mitophagy mechanisms contributes to the progression of CVDs (Tong et al., 2019; Tan et al., 2021). Briefly, the elimination of mitochondria by mitophagy requires the autophagy receptors to load mitochondrion to the autophagosome through light chain-3 (LC3) (Figures 3, 4). Mitochondria are then engulfed by the autophagosome, which merges with a lysosome to degrade its contents through the lysosomal enzymatic activity (Rüb et al., 2017). Mitophagy can be ubiquitin-dependent or receptor-dependent. Ubiquitin-dependent mitophagy mainly includes PINK1/Parkin-dependent pathways (Figure 4A). Receptor-dependent mitophagy includes Bcl-2 and adenovirus E1B19kDa-interacting protein 3 (BNIP3), FUNDC1, and lipid-dependent mitophagy pathways (Figure 4B).

#### 3.3.1 PINK1/Parkin-dependent mitophagy

During basal conditions, serine/threonine kinase PTEN-induced kinase 1 (PINK1) is constantly imported into the mitochondria as it bears a mitochondrial target signal (Lazarou et al., 2012). Once imported, PINK1 is cleaved by matrix processing peptidases, mitochondrial processing peptidase (MPP), and presenilins-associated rhomboid-like protein (PARL) to eliminate the mitochondrial target signal and its hydrophobic transmembrane helix within the transmembrane domain. After processing, PINK1 is released into the cytoplasm, where the UPS constantly degrades it. When mitochondrial damage occurs, PINK1 cannot be transported back to the IMM and accumulates on the OMM, unable to be cleaved by PARL, forming homodimers. Phosphorylation of PINK1 on S228 and S402 is required for its activation and engages Parkin to the OMM, inducing mitophagy (Lazarou et al., 2012; Okatsu et al., 2013; Yamano and Youle, 2013). Interaction of Parkin with PINK1 causes conformational changes that allow for its opening and phosphorylation on S65 by PINK1, simultaneously activating its E3 ubiquitin ligase activity (Ordureau et al., 2014; Pickrell and Youle, 2015) (Figure 4A). As a mechanism that further augments mitophagy, Parkin-mediated ubiquitination of OMM proteins serves as substrates for PINK1, which consequently recruits more Parkin (Nguyen et al., 2016). MFN2 can also be phosphorylated by PINK1 on the OMM and ubiquitinated by Parkin to prevent the fusion of damaged mitochondria with healthy ones (Chen et al., 2013) (Figure 4A). Mitochondria undergoing mitophagy are isolated through the interaction of autophagy-related gene (ATG) and LC3 proteins with a phagophore formation. First, the carboxyl-terminal region of pro-LC3 is processed by ATG4, a cysteine protease (Figure 4A). This results in glycine residues being exposed and the formation of LC3-I. Then, phosphatidylethanolamine (PE) binds to LC3-I via ATG7 and ATG3, forming LC-II as the first ubiquitin-like reaction. Following this reaction, together with ATG10, ATG7 deposits ATG12 to ATG5, which assembles the ATG12-5 complex. This complex later binds to ATG16, which endorses the recruitment of ATG8 with PE on the autophagosomes. Damaged mitochondria are identified through their LC3-interacting region (LIR), which ultimately leads to their engulfment by the autophagosome (Tanida et al., 2004; Nakatogawa, 2013; Dooley et al., 2014). Adaptor proteins sequestosome 1 (p62/SQSTM1), nuclear dot protein 52kDa (NDP52), optineurin (OPTN), and Tax-binding protein-1 (TAX1BP1) are crucial during the tethering of damaged mitochondria to the autophagosomal membranes via recognition of ubiquitinated proteins (Villa et al., 2018). The OMM proteins are eliminated when mitochondrial adaptor proteins contact LC3, which binds to the autophagosomal membranes. OPTN and NDP52 are found on the OMM of damaged mitochondria (Figure 4A). TANK-binding kinase 1 (TBK1) is responsible for the phosphorylation of OPTN and p62 at S177 and S403, respectively. These phosphorylations induce their tethering with ubiquitinated sites and LC3 (Figure 4A). Consecutively, OPTN and NDP52 induce autophagy via recruiting autophagy-related unc-51-like autophagy-activating kinase 1 (ULK1), which is phosphorylated and activated by AMP-activated protein kinase (AMPK) on S555 (Egan et al., 2011). Next, ULK1 recruits the Beclin-1 complex, which promotes the nucleation of phagophores, making autophagosome formation more efficient around the damaged mitochondria (Russell et al., 2013). This final step fully isolates targeted mitochondria inside the autophagosomes and triggers their fusion with lysosomes to form autolysosomes, leading to the degradation of the targeted mitochondria (Narendra et al., 2010; Weil et al., 2018; Yamano et al., 2020). Activation of mitophagy is not strictly dependent on AMPK activation, whereas ULK1 deficiency impairs PINK1/Parkin-dependent mitophagy (Vargas et al., 2019).

#### 3.3.2 BNIP3 and FUNDC1-dependent mitophagy

Bcl-2, BNIP3, and BNIP3-like (Nix) are all part of the Bcl-2 family of proteins and are constitutively expressed on the OMM (Ma et al., 2020) (Figure 4B). They have a prominent role in apoptosis but are also essential mitophagy receptors. These receptors are bound to the OMM and mainly interact with LC3 via the LIR motif to facilitate mitophagy, independent of the ubiquitin (Gustafsson Å and Dorn, 2019). It has been shown that BNIP3 and Nix are involved in the elimination of mitochondria in adult heart tissues (Lampert et al., 2019) and that hypoxic conditions can upregulate BNIP3 (Gálvez et al., 2006). Another mitophagy receptor, the FUN14 domain containing 1 (FUNDC1), mediates mitophagy (Figure 4B). This receptor is also relevant to hypoxia and ischemia, as hypoxic stimuli are linked with FUNDC1 dephosphorylation on its LIR via Phosphoglycerate mutase family Member 5 (PGAM5) to promote its binding with LC3 (Ren et al., 2020; Xiao et al., 2020). ULK1 is also known to phosphorylate FUNDC1 on its LIR motif to favor interaction with LC3 during mitophagy (Chen et al., 2014; Wu et al., 2014). Intriguingly, FUNDC1 has also been shown to interact with OPA1 and DRP1 to regulate mitochondrial dynamics during mitochondrial stress (Chen M. et al., 2016).

#### 3.3.3 Lipid-dependent mitophagy

Lipids, such as ceramide or cardiolipin, which can prompt mitophagy with their LIR motif, are present in the OMM. Ceramide is a sphingolipid synthesized by ceramide synthase (CeS). During ER stress, ceramides are translocated from the ER and accumulate on the OMM, triggering mitophagy (Chu et al., 2013). Accumulated ceramides on the OMM can bind LC3 through its ceramide-binding domain and promote the binding of Beclin1. Ceramide-mediated mitophagy also relies on DRP1, which initiates the recognition of ceramide in the OMM by the autophagolysosomes (Sentelle et al., 2012; Antón et al., 2016) (Figure 4B). Importantly, ceramides can build a channel with active BCL2-associated X protein (BAX) that allows the release of cytochrome c to initiate apoptosis. Another important lipid in mitophagy, cardiolipin, is a phospholipid synthesized by cardiolipin synthase (CRD1) in the mitochondria. Cardiolipin (CL) is present on mitochondrial membranes and cristae structures and is primarily found in the IMM (Daum, 1985). CL binds to OPA1 in physiological conditions to regulate OPA1 assembly during mitochondrial fusion and interacts with LC3 during mitochondrial stress (Liu et al., 2003). During mitochondrial damage, CL is transported from the IMM to the OMM (Chu et al., 2013). This movement allows CL to be exposed to the cytosol, bind to LC3, and serve as a recruitment site for adaptor proteins during mitophagy (Chu et al., 2013). Exposed CL preferentially associates with Beclin1 on the OMM during mitophagy and is crucial for phagophore formation (Huang et al., 2012). Notably, pro-IL-1α directly binds to CL in lipopolysaccharide (LPS)-treated macrophages through an LC3 binding domain while blocking mitophagy and activating NLRP3 inflammasome, further supporting the importance of CL in mitochondrial homeostasis (Dagvadorj et al., 2021).

### 3.4 Mitochondrial communication with other organelles

Mitochondrial networks cannot function separately from other subcellular compartments and rely heavily on inter-organelle communication. Mitochondria continuously coordinate with the nucleus, the ER, and other vesicular organelles (Lin et al., 2020). Importantly, inter-organelle communication of mitochondria and cytosolic organelles is crucial in the development and pathogenesis of several diseases. Most of the mitochondrial proteins are encoded in the nucleus and must be translocated into the mitochondria via transmembrane complexes (Pfanner et al., 2019). As mitochondria are self-monitoring organelles, any perturbations trigger retrograde signaling from mitochondria to the nucleus or other organelles to cope with the stress and reestablish proper mitochondrial functioning (Chowdhury et al., 2020; Vizioli et al., 2020; Booth et al., 2021).

#### 3.4.1 Mitochondrial unfolded protein response (UPR^mt^)

When mitochondrial protein import is dysfunctional, a signal from the mitochondria, called UPR^mt,^ is initiated and relayed to the nucleus. This signal activates the required transcription factors to cope with stress and induces a repair mechanism to normalize the mitochondrial protein import (Shpilka and Haynes, 2018). UPR^mt^ aims to optimize metabolism by blocking oxidative phosphorylation and the TCA cycle, decreasing the mitochondrial load and ultimately alleviating mitochondrial stress. Simultaneously, the expression of genes responsible for glycolysis and amino acid metabolism is augmented to provide alternative energy resources (Anzell et al., 2018). Aside from switching the reliance on specific energy resources, UPR^mt^ also promotes the expression of mitochondrial chaperones, anti-oxidative proteins, and proteases to cope with the accumulation of dysfunctional proteins (Zhu L. et al., 2021). This decreases the mitochondrial burden while enabling the proper processing of damaged mitochondrial proteins (Quirós et al., 2016). Impairment of OXPHOS machinery, mtDNA damage, disruption of mitochondrial protein synthesis, accumulation of misfolded or unfolded proteins in the mitochondria, or the buildup of ROS causes mitochondrial dysfunction. These events trigger UPR^mt^ (Qureshi et al., 2017). It has been shown that during mammalian UPR^mt^, transcription factors C/EBP homologous protein (CHOP), activating transcription factor 4 (ATF4), and ATF5 are activated, which are also involved in the unfolded protein response of the ER to alleviate ER stress caused by the accumulation of unfolded or misfolded proteins (Shpilka and Haynes, 2018; Yildirim et al., 2022). Activation of the kinases general control non-derepressible-2 kinase (GCN2), protein kinase RNA (PKR), PKR-like endoplasmic reticulum kinase (PERK), and heme-regulated inhibitor kinase (HRI) controls the phosphorylation of eukaryotic translation initiation factor 2 subunit 1 (eIF2**α**), which is the major player during organelle stress (Teske et al., 2013). Activated eIF2**α** inhibits global protein translation to reduce organelle stress, enhancing the expression of specific transcription factors like CHOP, ATF4, and ATF5. These transcription factors promote the expression of chaperones and proteases to cope with the UPR^mt^. Notably, the expression of ROS detoxifying enzymes, mitobiogenesis machinery, and mitochondrial import proteins are also enhanced to favor mitochondrial remodeling during stress (Qureshi et al., 2017).

#### 3.4.2 Calcium (Ca^2+^) signaling

Mitochondrial stress, which stems from the loss of mitochondrial membrane potential, releases calcium (Ca^2+^) from the mitochondria to the cytoplasm through voltage-dependent L-type Ca^2+^ channels. This increase in cytosolic Ca^2+^ triggers more Ca^2+^ to be released from the sarcoplasmic reticulum through the ryanodine receptor 2 (RYR2). Consequently, Ca^2+^ is cleared away from the cytoplasm via sarcoplasmic or ER resident ATPase (SERCA) family proteins, as well as solute carrier family 8 member A1 (SLC8A1) (Kho et al., 2012). During basal conditions, Ca^2+^ enters the mitochondria via the calcium uniporter protein (MCU) (Baughman et al., 2011). In addition, Ca^2+^ release occurs through the Ca^2+^ antiporter SLC8B1. Transient increases in Ca^2+^ levels can aid OXPHOS machinery. However, during mitochondrial membrane potential loss, free cytosolic Ca^2+^ activates calcineurin. In turn, the nuclear factor-κB (NF-κB) and nuclear factor of activated T cells (NFATC) are activated and translocated to the nucleus. This translocation stimulates the expression of genes responsible for Ca^2+^ storage or transport (Liu et al., 2017). Increased Ca^2+^ levels can activate various kinases like calcium/calmodulin-dependent protein kinase IV (CAMKIV), Ca^2+^-dependent protein kinase C, c-Jun N-terminal kinases (JNK), and p38 MAPK. These kinases fine-tune the mitochondrial adaptation to Ca^2+^ level fluctuations and metabolism, as well as cell proliferation and glucose metabolism (Giorgi et al., 2018).

#### 3.4.3 Intrinsic apoptosis pathway

During severe unresolved stress, like calcium overload, excessive ROS, or mtDNA damage, intrinsic apoptosis is initiated as mitochondrial homeostasis is jeopardized (Brumatti et al., 2010). Programmed cell death is orchestrated by pro-apoptotic proteins of the B cell lymphoma (BCL) 2 family. When apoptosis is triggered, BCL-associated X (BAX) and Bcl2 homologous antagonist/killer (BAK) are incorporated into the OMM and oligomerize, resulting in a pore formation on the mitochondria. This pore increases membrane permeability and promotes the release of pro-apoptotic factors (Lomonosova and Chinnadurai, 2008). Under physiological conditions, BAX is inactive and shuttles between the cytosol and the OMM, whereas BAK resides on the OMM and interacts with VDAC2 (Cheng et al., 2003; Schellenberg et al., 2013). Under apoptotic stimuli, BAX is no longer shuttled to the cytosol and together with BAK, gets activated via the pro-apoptotic Bcl2 homology-3 (BH3) domain-containing factors, BCL2 binding component 3 (also known as p53-upregulated modulator of apoptosis, PUMA), BCL2-interacting mediator of cell death (BIM), phorbol-12-myristate-13-acetate-induced protein 1 (NOXA) and BH3-interacting domain death agonist (BID) (Kuwana et al., 2005). Multiple pro-survival proteins counteract with the pro-apoptotic factors to reduce the permeabilization of the OMM, such as BCL2, BCL2 like 1 (BCL2L1 also known as BCL-XL), myeloid cell leukemia 1 (MCL1), BCL2 like 2 (BCL2L2, also known as BCL-W), and BCL2 related protein A1 (BCL2A1) (Moldoveanu et al., 2014). These factors reside on the OMM or ER and inhibit the binding of pro-apoptotic proteins (Czabotar et al., 2014). If the permeabilization of OMM is maintained, cytochrome c is released to initiate caspase-independent cell death. Notably, the release of cytochrome c causes apoptosome formation, a multi-protein structure composed of apoptotic protease-activating factor-1 (APAF-1), deoxyATP (dATP), and pro-caspase-9, where pro-caspase-9 gets activated (Chen et al., 2011). Consequently, caspases 3 and 7 are also cleaved and activated to promote cell death (Li et al., 1997; McArthur et al., 2018).

### 3.5 Mitochondria and inflammation

Mitochondrial apoptosis can produce type I interferon when caspases are inhibited (Rongvaux et al., 2014; White et al., 2014). In this context, mtDNA is released to the cytosol as a damage-associated molecular pattern (DAMP) during BAX/BAK-mediated pore formation and apoptosis (Cosentino et al., 2022). The presence of any DNA in the cytoplasm (e.g., pathogenic, nuclear, or mitochondrial) triggers a signal to activate 2′3′-cyclic GMP-AMP (cGAMP) synthase (cGAS), which detects cytosolic DNA. This detection allows cGAS dimerization and production of second messenger cGAMP to relay the message to and activate the stimulator of interferon genes (STING) (Diner et al., 2013; Sun et al., 2013). ER resident STING then shuttles to Golgi, activates TBK1, and gets phosphorylated by TBK1 (Zhang C. et al., 2019). Interferon regulatory factor 3 (IRF3) phosphorylation by active TBK1 promotes its dimerization and transport to the nucleus, which activates interferons and other cytokines (Tanaka and Chen, 2012). Oxidized mtDNA can exit the mitochondria through mPTP and VDAC, where oxidation promotes its fragmentation (Xian et al., 2022). In the cytosol, mtDNA fragments interact with NLRP3 to induce inflammasome activation and STING phosphorylation on S365, a pre-requisite for IRF3 activation (Chen Q. et al., 2016). Similarly, when released to the cytosol during programmed cell death, oxidized mtDNA binds to the NLRP3 inflammasome and activates it (Shimada et al., 2012). It has been recently shown that glycolytic enzyme Hexokinase 2 dissociation from VDAC on the OMM is essential for NLRP3 inflammasome activation. This dissociation results in Ca^2+^ uptake by mitochondria released from the ER and pore formation by VDAC oligomerization, allowing protein and mtDNA to exit from the mitochondria (Baik et al., 2023). Consequently, NLRP3 assembly is promoted and crucial during inflammatory responses.

Pathogenic or mitochondrial dsRNA in the cytoplasm is detected by the retinoic acid-inducible gene I (RIG-I) as well as melanoma differentiation-associated protein 5 (MDA5), which enables activation and aggregation of mitochondrial antiviral signaling protein (MAVS) on the OMM (Reikine et al., 2014). Accumulation of MAVS promotes IRF3 activation and stimulates the expression of antiviral response genes in the nucleus (Hou et al., 2011). Furthermore, bacterial infection can also activate an innate immune response that involves mitochondria. LPS or glycans on the bacterial wall can induce immune responses via ubiquitylation. This allows for the formation of pro-inflammatory ubiquitin linkages with the mitochondria and causes OMM permeabilization, enabling the formation of endolysosomes (Hamacher-Brady et al., 2014).

## 4 Mitochondrial dysfunction and CVDs

Mitochondrial dysfunction has been involved in the development of several CVDs (Killackey et al., 2020). CVDs are often associated with defective ETC machinery, excessive ROS production, impaired energetics, and MQC, as well as abnormal Ca^2+^ homeostasis (Forte et al., 2021). Notably, the dysregulation of proteins responsible for mitochondrial dynamics, mitophagy, or mitobiogenesis is closely linked to the advancement of CVDs (Chehaitly et al., 2022). Hence, understanding the contribution of mitochondrial health and normal functions to CVDs is crucial to develop novel and more efficient therapeutic approaches for these conditions. Here, we discuss various CVDs and the implications of mitochondrial dysfunction in this context (Table 1).

**TABLE 1:** Mitochondrial phenotype in CVDs. Overview of mitochondrial impairment during multiple CVDs.

Ischemia/Reperfusion	Hypertension	Atherosclerosis	Cardiac hypertrophy/heart failure	Diabetic cardiomyopathy	Genetic cardiomyopathies	Kawasaki disease
• Mitochondrial fragmentation	• Decreased endothelial NO levels	• Increased mitochondrial permeability	• Decreased mitochondrial mass	• Increased FA in cardiomyocytes	• mtDNA mutations in genes encoding for subunits of assembly factors Complex I, II or IV	• Increased NLRP3 inflammasome activation and IL1-β production
• Loss of mitochondrial membrane potential	• Elevated oxidative stress and mtROS	• Elevated mtROS	• Low ATP production	• Elevated mtROS production	• Impaired NADH-linked respiration	• Impaired clearance of damaged mitochondria *in vivo*
• Low ATP production	• ETC dysfunction	• NLRP3 inflammasome formation and impaired mitophagy	• Mitochondrial fragmentation	• Increased IMM potential and oxidative damage	• Downregulation in expression of genes related to ATP synthesis	• Decreased expression of autophagy-mitophagy related genes
• Increased mitochondrial membrane permeability	• Enhanced mitophagy during Ang-II induced model as protective mechanism	• Accumulation of damaged mitochondria	• Reduced ETC Complex I and IV activity	• Impaired mitochondrial dynamics and mitophagy	• Downregulation in mitochondrial mass and ETC super-complex formation	• Increased platelet subpopulations with different mitochondrial membrane potentials
• Ca^2+^ flux into mitochondria	• Reduced mitochondrial size		• Impaired mtDNA replication	• ETC dysfunction	• Dysregulated Ca^2+^ signaling	
• Increased mtROS						
• Impaired mitophagy						

mtROS, mitochondrial reactive oxygen species; ETC, electron transport chain; mtDNA, mitochondrial DNA; FA, fatty acid; IMM, inner mitochondrial membrane.

### 4.1 Ischemia/reperfusion (I/R) injury

Blood flow restoration of ischemic tissues or areas results in functional and structural tissue alterations, known as ischemia-reperfusion (I/R) injury. I/R injury usually stems from occlusion of coronary vessels, leading to tissue hypoxia and a deficiency in ATP levels (Peoples et al., 2019).

Mitochondria respond to cardiac tissue I/R injury, and a cascade of mitochondrial fragmentation and cell death, associated with elevated oxidative stress and loss of mitochondrial membrane potential is initiated upon reperfusion (Murphy and Steenbergen, 2008). Oxygen deprivation immediately results in mitochondrial dysfunction since heart tissues rely heavily on OXPHOS for their energy demand. *In vitro*, hypoxia/reoxygenation of cardiomyocytes revealed that these cells exhibit less mitochondrial fusion, lower ATP production, and higher membrane permeability (Liu and Hajnóczky, 2011). Furthermore, overexpression of mitochondrial fusion protein MFN2 or DRP1-dependent blockade of mitochondrial fission increased cell survival (Jahani-Asl et al., 2007). Mice overexpressing OPA1 display less severe I/R injury with better mitochondrial cristae remodeling (Varanita et al., 2015). Similarly, reduced OPA1 levels indicating a decrease in mitochondrial fusion during I/R injury in mice were rescued by melatonin treatment, which promotes AMPK signaling and diminishes caspase-9-dependent apoptosis (Zhang Y. et al., 2019). Supporting these findings, melatonin treatment also increased the expression of SIRT3 and the activity of the ROS-metabolizing enzyme SOD2 in cardiomyocytes during I/R by blocking the mitochondrial fission (Bai et al., 2021). Reduced infarct size is also observed in rats when mitochondrial fusion is promoted pharmacologically (Maneechote et al., 2019). Supporting these notions, cardiac-specific MFN1 knock-out mice are protected against excessive ROS production as these mice have a delayed mitochondrial permeability transition pore (mPTP) opening, resulting in increased cell survival (Papanicolaou et al., 2012). I/R injury also causes an increase in oxidative stress and promotes Ca^2+^ flux into the mitochondria, allowing a dysfunctional electrochemical gradient of IMM and impairing the ETC activity (Halestrap, 1999). Excess Ca^2+^ inside the mitochondria and elevated ROS levels cause the opening of the mPTP and render permeability of the IMM, resulting in depolarization, membrane potential loss, and cell death (Kwong and Molkentin, 2015). Similarly, overflow of Ca^2+^ into mitochondria due to mitochondrial calcium uniporter (MCU) activation is fundamental to I/R injury. Overexpression of Histidine triad nucleotide-binding 2 (HINT2) blocks MCU activity and alleviates Ca^+2^ burden and fragmentation of mitochondria in mice during I/R injury (Li et al., 2021).

Clearance of defective mitochondria by mitophagy is beneficial during I/R injury. Evidence indicates defective mitophagy, dysfunctional mitochondria, and myocardial impairment after I/R injury in DRP1 knock-out mice (Ikeda et al., 2015). Similarly, Parkin knock-out mice have exacerbated infarct size and dysfunctional mitochondria with impaired mitophagy, suggesting that by clearing defective mitochondria, mitophagy is beneficial during I/R injury (Kubli et al., 2013). Also, autophagy proteins such as Beclin 1, BNIP3, and FUNDC1 are implicated in the I/R injury (Georgakopoulos et al., 2017). FUNDC1 protects the heart by promoting mitophagy to eliminate damaged or non-functional mitochondria (Zhang et al., 2017). Furthermore, in a hypoxia model, the overexpression of necroptosis modulator receptor-interacting protein 3 (RIPK3) blocks the AMPK pathway and inhibits Parkin-dependent mitophagy in cardiomyocytes, leading to cardiomyocyte necroptosis and exacerbated cardiac damage (Zhu P. et al., 2021).

Data from FUNDC1 knock-out mice showed that this mitophagy factor regulates MQC, is essential for platelet activation, and ultimately decreases heart I/R injury (Zhang et al., 2016). In addition, an anti-diabetes drug, empagliflozin, has been reported to promote AMPK1/ULK1/FUNDC1-dependent mitophagy and attenuated mitochondrial dysfunction in both *in vitro* and *in vivo* I/R injury models (Cai et al., 2022). Upregulation of mitophagy and mitobiogenesis is observed in human cardiac tissues after cardiopulmonary bypass surgery, indicating that a balance between these two arms of MQC is crucial during surgery-induced I/R injury (Andres et al., 2017). These findings provide insight into how mechanisms of MQC regulate tissue homeostasis during I/R injury. However, therapeutic strategies to fine-tune mitochondrial dynamics during I/R injury need further investigation.

### 4.2 Hypertension

Hypertension, characterized by increased blood pressure and a decline in vascular function, is associated with inflammation, endothelial impairment, and mitochondrial dysfunction (Barki-Harrington et al., 2004; Lahera et al., 2017). During hypertension, oxidative damage in mitochondria is triggered by the activation of Angiotensin (Ang) II, which decreases endothelial NO levels and exacerbates vascular oxidative stress. ROS production increases during hypertension due to Ang II-mediated protein kinase-C activity and excess production of O_2_•^-^ and H_2_O_2_ (Doughan et al., 2008). Excessive ROS production may lead to mtDNA mutations and ETC machinery dysfunction, both contributing to hypertension progression (Griendling et al., 2021).

MQC also plays a pivotal role in the development of angiogenic function. It has been shown that the knock-down of MFN1, a protein involved in mitochondrial fusion, results in a less angiogenic response of endothelial cells to vascular endothelial growth factor (VEGF), induces endothelial NO synthase (eNOS) and decreases mitochondrial membrane potential (Lugus et al., 2011). Moreover, inhibition of mitochondrial fission by blocking DRP1 activity results in a blockade of apoptosis in an Ang II hypertensive rat model. In this context, SIRT1 is degraded by Ang II, resulting in p53 acetylation, which promotes DRP1 expression and cell apoptosis (Qi et al., 2018). Furthermore, genetic deletion of SIRT3 in mice leads to hypertension by inducing endothelial dysfunction, vascular inflammation, and hypertrophy. In contrast, global SIRT3 overexpression blocks Ang II-induced hypertension (Dikalova et al., 2020). Elevated FUNDC1-mediated mitophagy during hypoxic pulmonary hypertension induces hypoxia-inducible factor 1α (HIF1**α**) and pulmonary artery smooth muscle cell proliferation, leading to pulmonary vascular remodeling (Liu et al., 2022).

Mitophagy acts as a protective mechanism during hypertension and enhanced ATG-5-mediated mitophagy during Ang II-induced hypertension results in reduced levels of ROS and inflammation (Pan et al., 2012). Mitochondrial mass and structure are also affected during hypertension. Reduced mitochondrial size and osmotic swelling disrupt the mitochondrial OXPHOS efficiency (Ritz and Berrut, 2005). Cristae structure and ETC stability of mitochondria are jeopardized during hypertension, as it has been shown that there is a loss of CL, a crucial phospholipid for the membrane structure (Cogliati et al., 2013).

### 4.3 Atherosclerosis

Atherosclerosis is a progressive cardiovascular disease resulting in the formation of plaques containing inflammatory cells, the narrowing of arteries, and decreased blood flow, increasing the risks of cardiovascular complications, such as myocardial infarction and stroke. Plaque formation occurs via low-density lipoprotein (LDL) uptake in the arterial wall, leading to immune cell infiltration, inflammation, calcification, and vascular smooth muscle (VSMC) migration into the plaque (Gimbrone and García-Cardeña, 2016). Accumulation of oxidized LDL in the arterial wall can interfere with the proper functioning of mitochondrial respiration dynamics, cause the opening of mPTP and increase mitochondrial permeabilization, and promote ROS production (Khwaja et al., 2021). This eventually results in endothelial cell apoptosis and accelerates atherosclerosis. In addition, excess ROS production and mitochondrial dysfunction during atherosclerosis have been shown to activate NLRP3 inflammasome formation and boosts mitophagy (Jin Y. et al., 2022). Of note, loss of OGG1 induces oxidative mtDNA damage and subsequent NLRP3 inflammasome activation, exacerbating atherosclerosis in mice (Tumurkhuu et al., 2016). Importantly, depletion of an abundant cholesterol biosynthetic intermediate in coronary lesions, desmosterol, results in mtROS accumulation and NLRP3 inflammasome activation in macrophages, favoring atherosclerotic plaque formation (Zhang X. et al., 2021).

VSMCs can be activated by the platelet-derived growth factor (PDGF) during atherosclerotic plaque formation. MFN2 is less expressed in the atherosclerotic plaques, which favors mitochondrial fission. Inhibition of mitochondrial fission decreases VSMCs proliferation (Salabei and Hill, 2013). Similarly, inhibition of DRP1, a mitochondrial fission regulator, decreased endothelial impairment and atherosclerosis progression in apolipoprotein E (ApoE) knock-out mice, as well as VSMCs calcification (Rogers et al., 2017; Wang et al., 2017). Accumulation of p62 and LC3-II has been reported in cells from atherosclerotic plaques, which indicates impaired autophagy/mitophagy (Sergin et al., 2016). Decreased LC3-II levels are detected in patients with unstable atherosclerotic plaques, leading to reduced autophagy/mitophagy and defective clearance of damaged mitochondria and cell death in the arterial wall (Swaminathan et al., 2014). Supporting these findings, deleting the ATG-5 gene in mice, which is involved in autophagy, results in increased production of IL-1**β**, impaired cholesterol clearance, and increased atherosclerotic plaque formation (Razani et al., 2012). SIRT1 inhibition accelerates mouse atherosclerosis progression through the increased acetylation of ATG5 (Yang et al., 2017). Similarly, VSMC-specific deletion of ATG7 in *ApoE* ^*−/−*^ mice leads to impaired autophagic flux, accumulation of fragmented mitochondria, and inefficient OXPHOS activity with unstable atherosclerotic plaque formation susceptible to rupture (Nahapetyan et al., 2019). Furthermore, the importance of ER-mitochondria signaling in atherosclerosis progression has been shown through the modulation of the ER-resident kinase PERK. Lipid-activated PERK can activate Lon protease-1, resulting in mitochondrial PINK1 degradation and blockade of mitophagy. Inhibition of mitophagy increases ROS production due to the accumulation of damaged mitochondria and boosts inflammasome activation in macrophages, contributing further to atherosclerotic plaque formation (Onat et al., 2019). In addition, in the absence of a secreted protein known to participate in atherosclerosis, apolipoprotein A-I binding protein (AIBP), PINK1 is cleaved, and mitophagy is blocked, which also promotes atherosclerosis progression and plaque formation (Duan et al., 2022).

### 4.4 Cardiac hypertrophy and heart failure

Cardiac hypertrophy, a condition that results in the thickening of heart muscles, is an acute response to enhanced hemodynamic overload and mechanical stress on cardiac tissues. When prolonged, cardiac hypertrophy becomes pathological and can result in heart failure (Levy et al., 1990). Cardiac hypertrophy is associated with increased sarcomere production, thickening of the ventricular wall and induces changes in cardiac tissue gene expression, metabolism, and contractility (Schiattarella and Hill, 2015; Nakamura and Sadoshima, 2018). After hypertrophy, cardiomyocytes need more energy and increase mitochondrial mass at an early stage. However, this is followed by a decrease in mitochondrial mass and an impairment of cardiac contractility as the disease progresses (Zak et al., 1980; Goffart et al., 2004). Ultimately, the decline in mitochondrial mass reduces the production of ATP and diastolic dysfunction, causing heart failure (Flarsheim et al., 1996). BNIP3 expression increases in stressed cardiomyocytes, favoring mitochondrial fragmentation (Chaanine et al., 2016). Blocking mitochondrial fission through the deletion of DRP1 in mice leads to hypertrophy caused by norepinephrine signaling. On the other hand, inhibition of mitochondrial fusion by blocking MFN2 stimulates the hypertrophic response (Pennanen et al., 2014). Mitochondrial fusion is affected during cardiac hypertrophy, as reduced expression of MFN2 and OPA1 has been reported in cardiomyocytes in the context of heart failure (Chen et al., 2009). Furthermore, the activity of Complex I and IV from the mitochondrial ETC is also reduced during heart failure (Arbustini et al., 1998). mtDNA replication is also impaired in heart failure, causing a decrease in mtDNA-encoded proteins and mitochondrial biogenesis (Sheeran and Pepe, 2017). Interestingly, the deletion of OMA1, an inner mitochondrial membrane protease that determines cristae structure for optimal ETC functioning, alleviates myocardial damage by preventing cardiomyocyte death (Acin-Perez et al., 2018). The expression of dual-specificity tyrosine-regulated kinase 1B (DYRK1B) is increased in failing hearts of humans and mice and overexpression of this kinase impairs ejection fraction as well as cardiac fibrosis in mice by downregulating the mitobiogenesis regulator PGC1**α** (Zhuang et al., 2022). An important inflammatory response during pathological conditions is pyroptosis, a form of inflammatory cell death (Shi et al., 2015). During this process, NLRP3 inflammasome is induced, activating caspase-1, which cleaves gasdermin D (GSDMD) to permeabilize the cells, resulting in IL-1**β** and IL-18 secretion from the rupturing cells (Kayagaki et al., 2015; Shi et al., 2015). In mouse and human hypertrophic myocardia, it has been shown that there is an amplification of GSDMD that activates the mitochondrial STING axis, causing mitochondrial dysfunction and contributing to cardiomyocyte pyroptosis (Han et al., 2022). Notably, choline supplementation in rats alleviated cardiac hypertrophy by promoting UPR^mt^ through activating the SIRT3-AMPK axis as an adaptive response to cardiac dysfunction (Xu et al., 2019). Mitochondrial ROS scavenging is crucial to protect the cardiac tissues from oxidative damage (Xin et al., 2021). Recently, the deletion of mitochondrial peroxiredoxin 3 (Prdx3) in mice has been shown to cause cardiac hypertrophy and heart failure. In addition, the same study showed that Prdx3 interacts with PINK1 and blocks its proteolytic cleavage during mitophagy, playing a protective role during hypertrophy-induced mitochondrial dysfunction (Sonn et al., 2022). Of note, Parkin levels decline in hypertrophic cardiomyocytes, as well as mouse hearts, and overexpression of Parkin in mice blocked Ang-II-induced hypertrophy and improved heart function via boosting mitophagy, highlighting the importance of clearance of damaged mitochondria during cardiac hypertrophy (Sun et al., 2022).

### 4.5 Diabetic cardiomyopathy

Diabetic cardiomyopathy is a pathological process leading to changes in myocardial structure and function observed in patients with diabetes mellitus in the absence of other cardiac risk factors, such as coronary artery diseases or hypertension (Jia et al., 2018). Elevated levels of fatty acids in cardiomyocytes from diabetic patients can interfere with mitochondrial metabolism. In a healthy heart, ATP is produced by fatty acid oxidation and, to some extent, from carbohydrates (Duncan, 2011). Mitochondria and peroxisomes are responsible for the β-oxidation of fatty acids (FAO), a process that depends on oxygen availability. This process is not optimal when cardiac tissue needs high levels of ATP. In the context of diabetes, increased levels of fatty acids and ATP produced from the oxidation of FAO result in a loss of flexibility as to mitochondrial fuel sources (Duncan, 2011). Consequently, heart contraction efficiency starts to decline, causing a lack of blood flow and cardiomyopathy (Liesa and Shirihai, 2013). Nuclear receptor family PPARs are heavily involved in the pathogenesis of diabetic cardiomyopathy. Elevated levels of fatty acids inside cardiomyocytes interfere with FAO and lead to increased PPAR-γ expression and decreased PPAR-α expression (Wang L. et al., 2021). In line with this, an increase in fatty acids boosts ROS production by increasing IMM potential, resulting in oxidative damage and mitochondrial dysfunction (Schilling, 2015). Prolonged ROS levels increase and induce cardiomyocyte apoptosis, which is compensated by fibroblast recruitment. This changes the cellular composition of cardiac tissue, which can lead to heart failure (Flarsheim et al., 1996; Dabkowski et al., 2010).

Type 2 diabetic cardiomyopathy results in perturbations in the mitochondrial Ca^2+^ signaling (Dillmann, 2019). In a murine model of diabetic cardiomyopathy, mice fed with a high-fat and high-sucrose diet showed cardiac hypertrophy, dysfunction, and insulin resistance (Dia et al., 2020). This phenotype was associated with decreased interactions between IP3R and VDAC1, causing a decline in Ca^2+^ flux into the mitochondria (Dia et al., 2020). In a rat model of type 2 diabetes, the expression of secreted frizzled-related protein 2 (SFRP2), a protein involved in Wnt signaling and angiogenesis, is downregulated in rat cardiomyocytes and cardiac tissues. This was associated with a reduction in mitochondrial membrane potential and increased oxidative stress via the regulation of mitobiogenesis through the AMPK/PGC1α axis (Ma et al., 2021). While exercise in mice increased the expression of the mitochondrial deacetylase SIRT3 during diabetes-induced cardiac impairment and preserved cardiac functions by promoting the AMPK/SIRT3 pathway (Jin L. et al., 2022), SIRT3 deficiency exacerbates diabetic cardiomyopathy by activating NLRP3 and promoting the accumulation of mtROS (Song et al., 2021). The anti-diabetic drug empagliflozin has also been shown to inhibit cardiac dysfunction during diabetic cardiomyopathy by increasing the expression of *Nrf2* in type 2 diabetic mice. Additionally, in mice treated with empagliflozin, the expression of mitochondrial fission proteins was decreased with an upregulation of the fusion machinery, contributing to the recovery of cardiac tissues during this pathogenic condition (Wang J. et al., 2022). Notably, a decline in NAD^+^ to NADH ratio in diabetic mice is linked to the downregulation of SOD2, deficit in mitochondrial bioenergetics, and a concomitant increase in oxidative damage. Increasing NAD^+^ levels via nicotinamide phosphoribosyltransferase (NAMPT), the enzyme producing the intermediate for NAD^+^, relieved cardiac dysfunction in diabetic mice, showing the possible therapeutic potential of NAD^+^ supplementation in this disease (Chiao et al., 2021).

Mitochondrial dynamics are also impaired in type 2 diabetic cardiomyopathy. The decline in MFN1,2, OPA1, and DRP1 function has been implicated in the development of diabetic cardiomyopathy (Williams and Caino, 2018). A decrease in MFN2 expression, followed by impaired mitochondrial fusion, mitochondrial fragmentation, and related OXPHOS dysfunction, has been reported in diabetic cardiomyopathy (Montaigne et al., 2014). Perturbations in mitochondrial dynamics activate mitophagy and autophagy, the mechanisms of MQC. Previous data suggest that autophagy is blocked in type 1 diabetes, as shown by decreased levels of LC3, ATG5, and 12 in cardiomyocytes (Xu et al., 2013). Lower levels of PINK1 and Parkin are also reported in the same study, hinting at the suppression of mitophagy in the hearts of type 1 diabetic mice (Xu et al., 2013). In mouse models of type 1 diabetes, autophagy and mitophagy are augmented in cardiac tissues (Tao and Xu, 2020; Zhang M. et al., 2021). Furthermore, feeding mice with a high saturated fatty acid diet induces autophagy and cardiomyopathy (Russo et al., 2012). In addition, mice fed a high-fat diet developing diabetic cardiomyopathy show an initial peak of cardiac autophagy, which declines later. However, in this study, mitophagy activation was sustained for 2 months, leading to Parkin deficiency and impaired mitophagy, worsening the disease and suggesting a protective effect of mitophagy during the early stages of diabetic cardiomyopathy (Tong et al., 2019). Interestingly, the expression of TOM70, a mitochondrial outer membrane protein, is decreased in the heart tissues of diabetic mice (Wang et al., 2020). Overexpressing TOM70 rescues mitochondrial dysfunction, oxidative damage, and apoptosis in cardiac tissues, indicating that TOM70 could be a therapeutic target in treating diabetic cardiomyopathy (Wang et al., 2020).

### 4.6 Genetic cardiomyopathies

Genetic cardiomyopathies arise from chromosomal abnormalities interfering with the normal function of the heart (McKenna et al., 2017). Hypertrophic cardiomyopathy is the most prevailing, affecting 1 in 500 people (Tuohy et al., 2020). It is usually caused by autosomal dominant mutations of genes that code for sarcomere proteins and mutations of mitochondria-related genes (Teekakirikul et al., 2019). Arrhythmogenic right ventricular cardiomyopathy is a familial heart disease that manifests by mutations of desmosomal proteins, which are intermediate muscle tissue filaments (Krahn et al., 2022). Consequently, ventricular wall thinning and ballooning occur in affected patients (Shah et al., 2023). Another congenital cardiomyopathy is left ventricular non-compaction, which is initiated during the embryonic stage and perturbs the normal development of the heart muscle (Singh and Patel, 2023). In addition, restrictive cardiomyopathy is characterized by the restriction of the left ventricle, causing a significant increase in ventricular pressure and myocardial stiffness (Rapezzi et al., 2022). Finally, dilated cardiomyopathy is manifested with enlarged ventricles, causes systolic dysfunction, and is inherited in an autosomally dominant manner (De Paris et al., 2019).

Genetic cardiomyopathies are mitochondrial diseases related to either disruption in ETC complex subunits or factors involved in their assembly, mitochondria encoded tRNAs, rRNAs, and proteins responsible for mtDNA maintenance and CoQ_10_ synthesis (Yamada and Nomura, 2021). These cardiomyopathies are often associated with mutations in mtDNA encoded Complex I subunit genes NADH-ubiquinone oxidoreductase chain 1 and 5 (MT-ND1 and 5) and nuclear-encoded Complex I subunit genes such as NADH: ubiquinone oxidoreductase core subunits S2 (NDUFS2), V2 (NDUFV2) or A2 (NDUFA2). Similarly, mutations in nuclear factors involved in Complex I assembly such as acyl-CoA dehydrogenase family member 9 (ACAD9) and NADH: ubiquinone oxidoreductase complex assembly factor 1 (NDUFAF1), are also associated with the development of genetic cardiomyopathies (Fassone and Rahman, 2012; Brecht et al., 2015; Dubucs et al., 2023). All subunits of Complex II are nuclear-encoded, and mutations of subunits A, D (SDHA and SDHD), as well as Complex II assembly factor 4, have been reported in patients with hypertrophic, dilated, and non-compaction cardiomyopathies (Jain-Ghai et al., 2013; Alston et al., 2015; Wang X. et al., 2022). Likewise, mutations in mtDNA encoded cytochrome b gene (MTCYB) have also been identified in patients with hypertrophic and dilated cardiomyopathy (Hagen et al., 2013; Carossa et al., 2014). Supporting these findings, mutations in Complex IV subunit genes COX6B1, MTCO2, and MTCO3 have been associated with dilated and hypertrophic cardiomyopathies (Abdulhag et al., 2015; Yang et al., 2022). Moreover, a possibly pathogenic m.9856T>C (Ile217Thr) mutation in MTCO3 has been identified in a left ventricular non-compaction cardiomyopathy patient, together with a significant decrease in mtDNA copy number in the patient group compared with controls (Liu et al., 2013). Interestingly, hypertrophic cardiomyopathy has been associated with m.2336T>C homoplasmic mutation of mitochondrial 16S rRNA gene (MTRNR2), and restrictive cardiomyopathy has been linked to m.1555A>G mutation of the mitochondrial 12S rRNA gene (MTRNR1) (Liu et al., 2014; Li et al., 2019). Furthermore, c.467T>G and c.950C>G mutations in mitochondrial ribosomal proteins L44 and L3 (MRPL44 and 3) have also been reported in hypertrophic cardiomyopathy, respectively (Galmiche et al., 2011; Distelmaier et al., 2015). Mutation in genes involved in CoQ_10_ biosynthesis, specifically COQ2, 4, and 9, have been reported in patients with hypertrophic cardiomyopathy (Doimo et al., 2014; Desbats et al., 2015; Mantle et al., 2023).

Mitochondrial dysfunction in hypertrophic cardiomyopathy has recently been associated with impaired NADH-linked respiration, as well as fatty acid oxidation in septal myectomy tissues from patients, due to mitochondrial fragmentation, which was rescued by NAD^+^ supplementation (Nollet et al., 2023). Interestingly, cardiac-specific deletion of cytochrome c assembly factor *Cox10* resulted in mitochondrial cardiomyopathy in mice, leading to OXPHOS defects. Furthermore, the activation of mitochondrial peptidase OMA1 appears protective during mitochondrial cardiomyopathy (Ahola et al., 2022). A multi-omics study investigating healthy controls and patients with hypertrophic cardiomyopathy indicates a global downregulation in the expression of mitochondrial genes involved in ATP synthesis, coupled with mitochondrial damage manifested by low cristae density and less oxidative phosphorylation (Ranjbarvaziri et al., 2021).

During dilated cardiomyopathy, mitochondria numbers first increase to compensate for the energy deficit and then decrease over time, which results in less ATP production, deficiencies in cardiac contractility, and more ROS production (Goffart et al., 2004; Ramaccini et al., 2020). It has been reported that cardiomyocytes lacking the circadian rhythm activation factor basic helix-loop-helix ARNT like 1 (BMAL1) gene exhibit a dilated cardiomyopathy-like phenotype with reduced expression of mitochondrial BCL2 interacting protein 3 (BNIP3) protein, resulting in defective mitophagy and cardiomyocyte function (Li et al., 2020). Additionally, in a doxorubicin-induced mice model of dilated cardiomyopathy, increased expression of full-length SIRT3 prevented cardiac dysfunction by blocking superoxide generation (Tomczyk et al., 2022).

Mitochondrial dysfunction also contributes heavily to arrhythmogenic cardiomyopathy, as there are deficits in intracellular Ca^2+^ dynamics and ion channel functioning (Kim et al., 2019). Connexin 43 (Cx43), found on IMM of subsarcolemmal cardiomyocytes for regulation of ATP synthesis and respiration, is shown to decrease in arrhythmogenic cardiomyopathy patients, resulting in dysfunctional cardiomyocyte excitability (Noorman et al., 2013).

Pathogenic variants of desmin, a crucial component of the intermediate filaments of cardiac or skeletal muscle cells, can cause desminopathy, characterized by cardiomyopathy and skeletal myopathy (van Spaendonck-Zwarts et al., 2011). Dilated, arrhythmogenic, and restricted cardiomyopathy is often linked to desminopathy (Taylor et al., 2007; Otten et al., 2010; Lorenzon et al., 2013). A recent study identified a p.(S57L) mutation in desmin, which was associated with a striking downregulation of mitochondrial proteins and bioenergetics, coupled with a decrease in ETC Complex I + III + IV super-complex formation (Kubánek et al., 2020). In addition, VDAC1 has been shown to aggregate on muscle fibers together with apoptotic proteins, both in patients with desminopathy and in the rat model of this disease (Li et al., 2016).

### 4.7 Kawasaki disease (KD)

KD is an acute febrile systemic vasculitis of unknown etiology, affecting infants and young children, and is the leading cause of acquired heart disease in developed countries (Kawasaki et al., 1974; Newburger et al., 2016; McCrindle et al., 2017). Coronary arteritis and coronary artery aneurysms (CAAs) occur in approximately 25% of untreated children, which is reduced to 3%–5% with high-dose intravenous immunoglobulin (IVIG) treatment (Burns et al., 1998; Tremoulet et al., 2008). Transcriptome analysis of whole blood from KD patients as well as cardiovascular tissues collected from *Lactobacillus casei* cell wall extract (LCWE)-injected mice, an experimental mouse model mimicking KD vasculitis, showed increased expression of genes associated with IL-1β and the NLRP3 inflammasome pathway (Hoang et al., 2014; Lee et al., 2015; Wakita et al., 2016; Porritt et al., 2021). Supporting NLRP3 activation, high levels of serum concentration of the pro-inflammatory cytokines IL-1**β** and IL-18 have been reported in the plasma of children with KD during the acute phase of the disease (Alphonse et al., 2016). Blocking the IL-1 pathway in two different experimental mouse models of KD, the LCWE- and the *Candida albicans* water-soluble fraction (CAWS), either pharmacologically or using knock-out mice, significantly decreases vasculitis severity (Lee et al., 2012; Lee et al., 2015; Anzai et al., 2020; Porritt et al., 2020). Several case studies have also demonstrated the efficacy of Anakinra, an IL-1 receptor antagonist, in treating IVIG-resistant KD patients (Cohen et al., 2012; Shafferman et al., 2014; Guillaume et al., 2018; Blonz et al., 2020; Koné-Paut et al., 2021).

Mitochondrial dysfunction and impaired autophagy and mitophagy can trigger NLRP3 inflammasome activation (Zhang N. P. et al., 2019). Impaired clearance of damaged mitochondria has been reported in the LCWE-induced murine model of KD vasculitis, shown by a decreased autophagic flux and blockade of autophagy by mTOR pathway activity, contributing to NLRP3 inflammasome activation and ROS accumulation (Marek-Iannucci et al., 2021). The expression of *LC3B*, *BECN1,* and *ATG16L1* mRNA is reduced in whole blood of KD patients compared with febrile and healthy controls, while IVIG treatment increases the expression of these autophagy-related genes (Huang et al., 2020). However, low expression of *ATG16L1* mRNA persists in patients developing coronary artery lesions, further indicating that the autophagy/mitophagy pathway is disrupted during KD and could be a possible therapeutic target (Huang et al., 2020). Statins, specifically atorvastatin, have been used to treat KD patients developing giant coronary aneurysms to improve endothelial cell homeostasis (Niedra et al., 2014). Studies have shown that statins also block NLRP3 inflammasome activation by promoting autophagy/mitophagy (Andres et al., 2014; Peng et al., 2018; Tremoulet et al., 2019). In addition, alleviating ER stress in mice by deletion or pharmacological inhibition of ER resident endoribonuclease IRE1 also reduces LCWE-induced KD vasculitis as well as caspase-1 activity and IL-1**β** production, highlighting the importance of ER stress pathways in KD progression (Marek-Iannucci et al., 2022). Serum levels of β-hydroxy-γ-trimethylammonium butyrate (carnitine), an amine that transfers long-chain fatty acids across the IMM for β-oxidation, is found to be higher in IVIG non-responsive KD patients, hinting towards altered mitochondrial fatty acid metabolism and mitochondrial fuel dependency (Muto et al., 2022).

Increased platelet count is commonly reported in KD patients in the second to third week of disease onset (Levin et al., 1985; Arora et al., 2020), as well as in the LCWE-induced mouse model of KD (Kocatürk et al., 2023). Two separate platelet sub-populations co-exist in the peripheral blood of KD patients (Pietraforte et al., 2014). The first one is characterized by mitochondrial membrane potential loss, whereas the second one exhibits mitochondrial membrane potential hyperpolarization. These platelet sub-populations may potentially influence inflammasome responses, the redox state, and calcium balance in the development of the KD vasculitis (Pietraforte et al., 2014). Therefore, further studies are warranted to investigate the potential role of mitophagy and autophagy on the development of KD cardiovascular lesions.

## 5 Therapeutic strategies targeting mitochondria in CVDs

Modulating mitochondrial function and reducing mitochondrial oxidative stress could be a therapeutic target to prevent and/or ameliorate CVDs. Mitochondria-targeted antioxidant MitoQ, is a synthetic ROS-scavenger that mimics the activity of mitochondrial CoQ_10_ (Murphy and Smith, 2007). MitoQ treatment is available orally, and safe (Rossman et al., 2018; Oliver and Reddy, 2019), and its uptake by the mitochondria is dependent on membrane potential (James et al., 2007). MitoQ alleviates doxorubicin-induced cardiomyopathy in rats (Chandran et al., 2009) and promotes vascular endothelial function in older patients by reducing age-related ROS accumulation (Rossman et al., 2018). Similarly, another mitochondria-targeted ROS scavenger mimetic, mitoTEMPO, decreases mPTP opening to prevent mitochondrial apoptosis (Liang et al., 2010), and reduces diabetic cardiomyopathy in mice (Ni et al., 2016).

Mitochondria generate ATP through OXPHOS, and byproducts of the TCA cycle, such as NAD^+^ in its reduced form, NADH, contribute as a primary source of electrons to this process (Figure 2). NAD^+^ is a cofactor involved in cellular metabolism and mitochondrial fitness, and reduced NAD^+^ levels are associated with dysfunctional mitochondria (Srivastava, 2016). Therefore, targeting and boosting NAD^+^ could improve mitochondrial function in CVDs. Dietary supplementation with NAD^+^ precursor nicotinamide mononucleotide (NMN) can restore mitochondrial function in murine models of heart failure (HF) (Wang Y. J. et al., 2021), dilated cardiomyopathy (Martin et al., 2017) and I/R injury (Yamamoto et al., 2014). NMN has also been shown to promote autophagy and to decrease hypertension-mediated stroke in rats (Forte et al., 2020). Oral supplementation of mice with nicotinamide riboside (NR), another NAD^+^ intermediate, also boosts NAD^+^ and attenuates heart failure and dilated cardiomyopathy (Diguet et al., 2018). Chronic supplementation with NR is well tolerated and effectively boosts NAD^+^ levels in healthy middle-aged and older adults (Martens et al., 2018).

Another important regulator of autophagy/mitophagy, SIRT1, an NAD-dependent deacetylase, is non-specifically activated by resveratrol, a natural polyphenol produced in plants (Baur and Sinclair, 2006), as well as synthetic sirtuin-activating compounds such as SRT1720 (Bonkowski and Sinclair, 2016). These molecules have been shown to limit disease severity in multiple rodent CVD models, including Ang-II-induced atherosclerosis (Chen et al., 2015), cardiac hypertrophy (Chan et al., 2008), and diabetic cardiomyopathy (Sulaiman et al., 2010). Boosting NAD^+^ levels activates sirtuins and improves mitochondrial function (Kane and Sinclair, 2018).

The AMPK pathway is another crucial regulator of mitochondrial biogenesis and mitophagy. As mentioned above, AMPK activates PGC1α to promote mitochondrial biogenesis (Kukidome et al., 2006; Jäger et al., 2007). Metformin activates AMPK and promotes survival during heart failure in mice (Gundewar et al., 2009). Resveratrol activates mitochondrial biogenesis via AMPK and downstream PGC1α and TFAM (Lagouge et al., 2006). Notably, resveratrol improves cardiac function in mice and rats with hypertension (Rimbaud et al., 2011; Magyar et al., 2012). Caloric restriction and exercise can also induce the AMPK pathway, promote mitophagy, and prevent inflammatory response (Laker et al., 2017; Chen et al., 2020). Furthermore, inhibition of mTOR with rapamycin induces mitophagy in mice cardiomyocytes and increases lifespan (Wei et al., 2015; Yang et al., 2019). Promising developments in approaches/drugs improving mitochondrial homeostasis in cardiac tissues are crucial for discovering novel therapeutic agents, and future studies are needed to explore better targets in the context of CVDs.

## 6 Conclusion

Proper mitochondrial functioning is essential for tissue homeostasis, particularly in cardiac tissues with high demand for energy, such as the heart. Therefore, MQC mechanisms are central to maintaining cardiac homeostasis via the clearance of damaged mitochondria. Adapting the mitochondrial network to stress allows the heart to preserve its function during disease. The pathology of CVDs is highly connected to mitochondrial structure, membrane potential, dynamics, and clearance. Maintaining the health of the mitochondrial network via MQC is necessary for the outcome of CVDs. Therefore, mitochondria-targeting agents are critical therapeutic options to prevent or alleviate mitochondrial dysfunction during cardiac pathology. Current studies have shown that cardiac tissue protection can be achieved through mitophagy, ROS detoxification mechanisms, replenishing mitochondria pool, and inter-organelle communication. Targeting these MQC pathways is pivotal in discovering new treatments for CVDs in the future.
